# Application of Personalized Education in the Mobile Medical App for Breast Self-Examination

**DOI:** 10.3390/ijerph19084482

**Published:** 2022-04-08

**Authors:** Joanna Błajda, Edyta Barnaś, Anna Kucab

**Affiliations:** Institute of Health Sciences, Medical College of University of Rzeszów, Kopisto 2a, 35-959 Rzeszow, Poland; ebarnas@interia.eu (E.B.); apieniazek@ur.edu.pl (A.K.)

**Keywords:** mobile apps, breast self-examination, breast cancer

## Abstract

Introduction. Mobile apps are considered intelligent tools useful in various areas of public health. The social dimension of breast cancer and the current epidemic situation require tools that may increase knowledge and improve the skills in the field of breast self-examination. The study aims to assess the use of personalized education based on algorithms with conditions in the mobile medical app for breast self-examination. Materials and methods. In total, 500 women from the Podkarpackie Province were enrolled in the study, which was a representative group for the inhabitants of this province. The subjects were randomly divided into two groups (group I: the study group including 250 people; group II: the controls of 250 people). The study group was subjected to intervention, which was personalized education on breast cancer. The method was a proprietary mobile medical app based on algorithms with conditions. The study was carried out from March 2018 to February 2019. Results. The majority of women, 77.8% (N = 389), were under 30 years of age. Only a small amount of the breast area was marked in the tactile test in both groups. In the study group, the average number of selected points was 14.86 (7.43% of the area to be examined), while in the control group it amounted to 9.14 (4.57%). The area most commonly examined in Test I in both groups was the central area of the mammary gland with the nipple. After the intervention in Test II, women from the study group marked a significantly greater area in the tactile test than women from the control group (χ^2^ = 99.733; *df* = 6; *p* < 0.0001). The mean result in the study group was 22.10, while in the control group it amounted to 9.10. It was found that the breast area marked in both tests depended solely on the women’s knowledge about breast cancer (*p* < 0.001). It was also found that the higher the risk of developing breast cancer, the more points in Test I were indicated by the women in the tactile test (*p* = 0.0122). Conclusions. Educational mobile medical apps for breast cancer prevention may help to deal with breast cancer, which is an important public health issue. It is also important to broaden the possibilities of medical apps for breast self-examination with elements verifying the skills of the three-stage compression of the examined breast.

## 1. Introduction

The dynamic development of mobile technology, including medical apps, facilitates changes in health care, education and research [1]. The current epidemic situation worldwide has also increased the demand for remote access to medical and educational services. Perspective applications and technological solutions used in mobile medical apps allow us to recognize them as intelligent tools. Numerous scientists draw attention to their already significant role and perspective implications in boosting knowledge, awareness and skills in the field of health behaviors, including the prevention and early detection of breast cancer [2]. Online app stores offer access to numerous interesting medical apps related to the broadly understood issue of breast cancer. High demand for this kind of app stems from the fact that breast cancer has been a serious global problem for many years. Breast cancer is the most common cancer in women worldwide. It develops in women from every country of the world at any age after puberty but with increasing rates in later life [3]. An important role in solving this issue may be played by health education and learning the correct technique of breast self-examination.

### 1.1. Aim

The study aims to assess the use of personalized education based on algorithms with conditions in the mobile medical app for breast self-examination.

### 1.2. Material

The study involved a group of 500 women from the Podkarpackie Province (south-eastern part of Poland). which was randomly divided into two equal groups (the study group and the controls). The study group consisted of 250 individuals subjected to an intervention. The control group included 250 women not subjected to any intervention in the form of personalized education.

The target group of women was estimated in relation to the data on the number of women in the Podkarpackie Province from the Local Data Bank of the Central Statistical Office website. It was established that in 2016, 895,329 women aged 18+ lived in the Podkarpackie Province. The above data were used as input to determine the percentage of the minimum sample size with a confidence interval of 0.95% and a maximum error of 0.05. The minimum percentage of the sample size was 384 people, and it was increased to 500 people.

The cohort was randomly divided. The software used in the app alternately assigned subjects to the groups. The first person using the app was assigned to the study group, then all people using the app in the order of odd numbers were subjected to the intervention. On the other hand, the second person and the rest of the group in the order of even numbers were included in the control group, which was not subjected to any intervention. Inclusion criteria for the study: female gender, age ≥18 years, informed consent to participate in the study, a resident of the Podkarpackie Province and have a mobile device with Internet access. Exclusion criteria: refusal to participate in the study, place of residence outside the Podkarpackie Province, male gender, age <18 and have a mobile device without Internet access. The study was carried out from March 2018 to February 2019. The study protocol was approved by the Bioethics Committee No. 4/06/2016 operating at the University of Rzeszów.

#### Intervention

**Figure 1:**
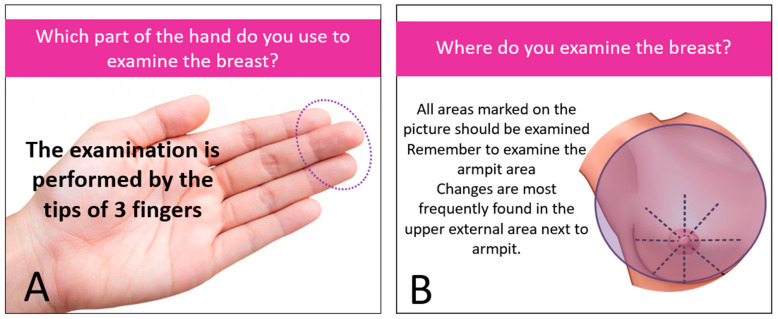
Personalized education. (**A**) example no.1 (**B**) example no. 2.

**Figure 2:**
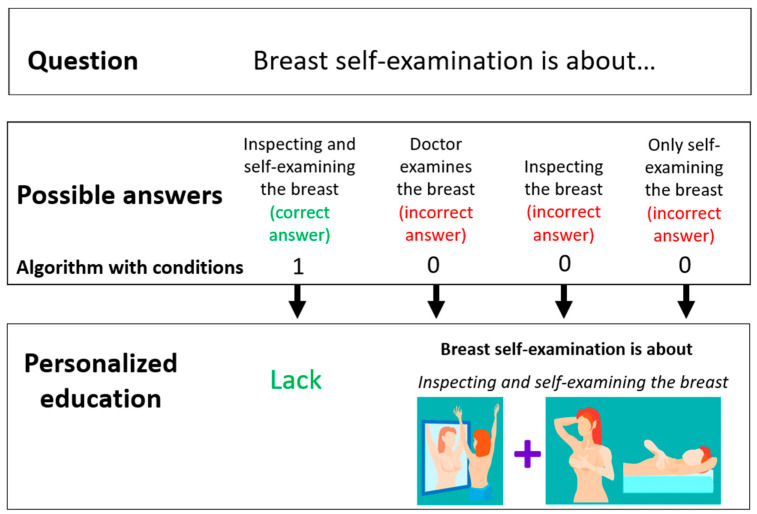
Principles of the intervention operation based on one of the questions.

The intervention (personalized education) was dedicated only to the study group and took place immediately after the completion of the first stage of the study. Based on her answers provided in the proprietary questionnaire “Check your knowledge about breast cancer”, the respondent received a result assessing the level of her knowledge about breast cancer prevention. The subject received comprehensive, personalized information on breast cancer prevention and breast self-examination. They were individually tailored, adequate to the demonstrated knowledge deficit, as shown in Figure 1 The principles of the intervention are presented on the example of one of the questions, as shown in Figure 2.

## 2. Method

**Figure 3:**
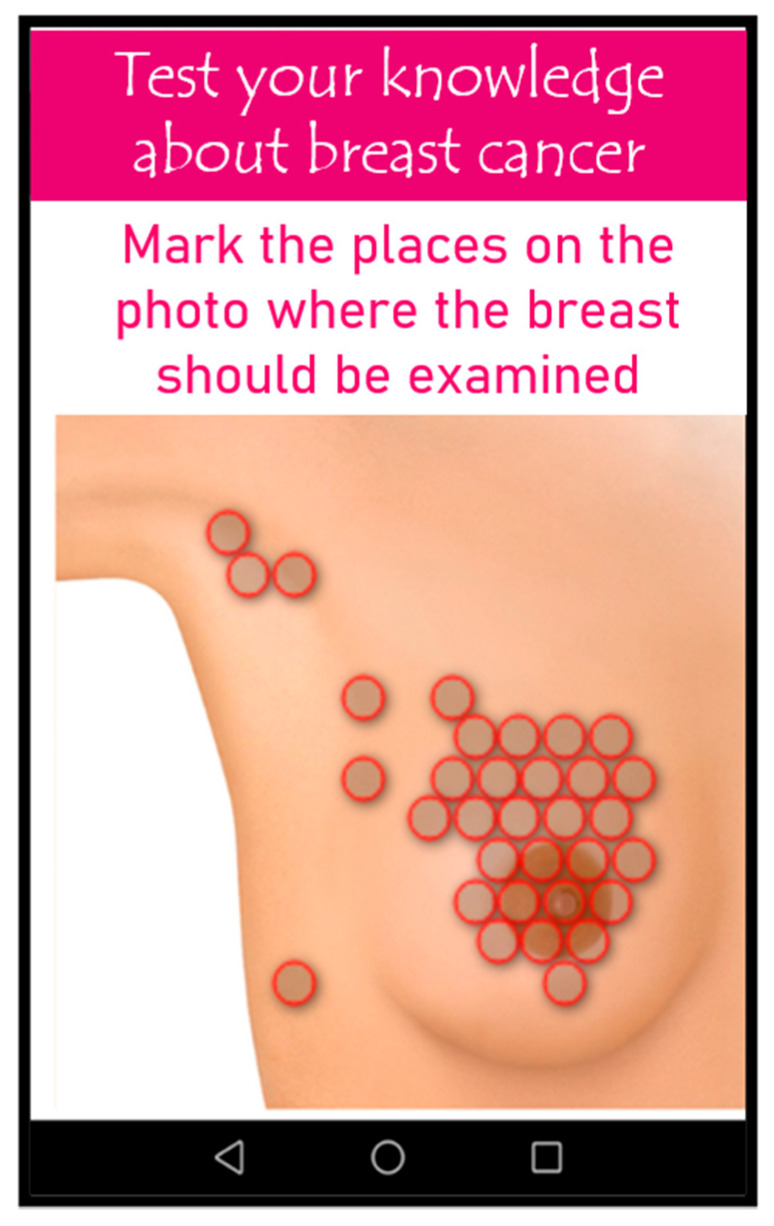
The image of the proprietary interactive tactile test—breast self-examination.

For the purposes of the study, a proprietary mobile app was developed—“Exam oneself”. Access to the app was possible using any mobile device with Internet access: a tablet or a smartphone that supports the Android system. The app used both standardized and proprietary data collection tools:

- Proprietary interactive tactile test: The purpose of which was to evaluate the technique of breast self-examination. The test included a graphic model of the breast. Users were asked to palpate the graphically depicted breast. On the surface of the illustration, there were 200 mapped points closely adjacent to one other, which cover the entire surface of the mammary gland. These points were assigned values in the software (0—if the person did not mark the point, 1—if the point was marked). Additionally, the selected area on the graphic model changed its color when touched. As an illustration, a graphic model of the breast with no apparent breast cancer symptoms was intentionally used so as not to suggest any changes that required examination. The following parameters were assessed in the test: percentage of the selected area, places most frequently marked/omitted, places on the model from which the study was started (Figure 3). The tactile test was developed by a medical professional with experience in breast self-examination and by a programming specialist. The appearance of the test and the principles of operation were the original idea of a medical specialist. The software used in the tactile test was developed by a programmer based on the guidelines of a medical specialist. A medical specialist supervised the development of the software and tested the software at various stages of its development, as well as interpreted the obtained test results.
- Proprietary questionnaire: check knowledge about breast cancer.
- Standardized questionnaire: Generalized Self-Efficacy Scale (GSES).

The algorithm with conditions was used in the proprietary mobile app of “Exam oneself”. The app contained a conditional instruction with assigned points. Personalized education was closely related to the proprietary questionnaire “check your knowledge about breast cancer”. If the application user chose the wrong answer in the proprietary questionnaire “check your knowledge about breast cancer”, she scored 0 points. Being awarded 0 points was a condition for obtaining information on an individual question. On the other hand, when a person gave a correct answer to a given question and obtained 1 point—the condition was not met, and in this case, educational information on this topic did not appear. The knowledge test included many questions about the breast self-examination technique. The proprietary questionnaire, “check knowledge about breast cancer”, and the interactive tactile test for breast self-examination are included in the Supplementary Materials.

### 2.1. Study Design

**Figure 4:**
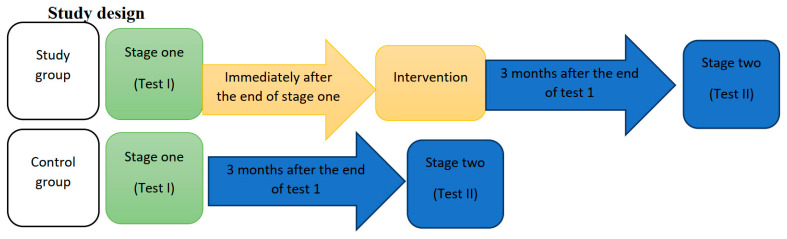
Study Design.

Test I concerned both the study group and the control group, and it contained the following questionnaires: the Proprietary questionnaire—“test your knowledge about breast cancer”, the interactive tactile test—“breast self-examination” and the Generalized Self-Efficacy Scale (GSES)—measuring the strength of an individual’s overall belief in the effectiveness of dealing with difficult situations and obstacles (Figure 4).

Test II concerned both the study group and the control group, and it was carried out 3 months after completing Test I. The measurement consisted in repeating the proprietary questionnaires—check knowledge about breast cancer, and the proprietary interactive tactile test (Figure 4).

### 2.2. Statistical Analysis

The collected data were subjected to statistical analysis R version 3.6.0, by means of PSPP and MS Office 2019 software. The significance level was adopted at *p* = 0.05. Accordingly, the results of *p* < 0.05 indicated the existence of significant relationships between the variables. Parametric tests (student’s *t* test or ANOVA) or their non-parametric equivalents (Mann–Whitney U test or Kruskal–Wallis test) were used to analyze quantitative variables by a group. The analysis of quantitative variables (i.e., expressed in number) was performed by calculating the arithmetic mean (x), standard deviation (SD), median (Me), minimum (min) and maximum (max). The analysis of qualitative (i.e., non-numeric) variables was performed by calculating the number and percentage of occurrences of each value. Multivariate linear regression was used in the study. Multivariate (or multivariate) linear regression is an extension of simple regression with a single predictor. Multivariate regression allows us to evaluate how several explanatory variables affect the explained variable. The analyzed dependent variables were the number of marked points in the tactile test.

## 3. Results

The majority of the surveyed women were 30 years or below (389 respondents, i.e., N = 77.8%). The respondents from two groups more often lived in rural areas than in cities. The total number of rural residents was 53.0%, i.e., N = 265. Most of the women (N = 297, i.e., 59.4%) had secondary education. Overall, 34.2% of the subjects (N = 171) had higher education. People not working and studying at the same time constituted the most numerous group (N = 203, i.e., 40.6%). In total, 27.0% of the respondents (N = 135) did mental work, while 20.0% of the women (N = 100) performed physical work and 12.4% of the respondents (N = 62) did not work (Table 1).

### 3.1. The Results of Test I of the Proprietary Interactive Tactile Test

When assessing the number of points marked on the breast model, the answers were classified into four ranges (0–3 points, 4–6 points, 7–13 points and over 13 points). In the study group, 30.4% (N = 76) of the respondents indicated more than 13 (out of 200 possible points), while in the control group only 18.4% (N = 46) of women obtained that result (χ^2^ = 14.535, *df* = 3, *p* = 0.002) (Table 2).

**Figure 5:**
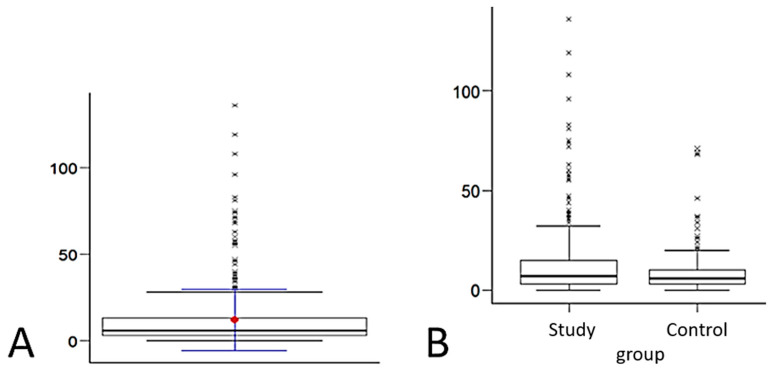
Number of points marked in the tactile test—Test I: (**A**) mean number of points and (**B**) distribution of variables.

During Test I, the women marked on average 12 points (6% of the breast area). Their number ranged from 0 to 136 points (Table 3, Figure 5A).

Half of the study group obtained a result not lower than Me = 7.00. The minimum result among the study group was Min = 0.00, while the maximum score was Max = 136.00.

Half of the control group obtained a result not lower than Me = 5.00. The minimum score in the control group was Min *=* 0.00, and the maximum score was Max = 71.00 (Table 4).

The study group achieved a result significantly higher (*p* < 0.05) than the control one in terms of breast examination in Test I—the proprietary interactive tactile test “breast self-examination”. The distribution of variables is presented in Figure 5B.

**Figure 6:**
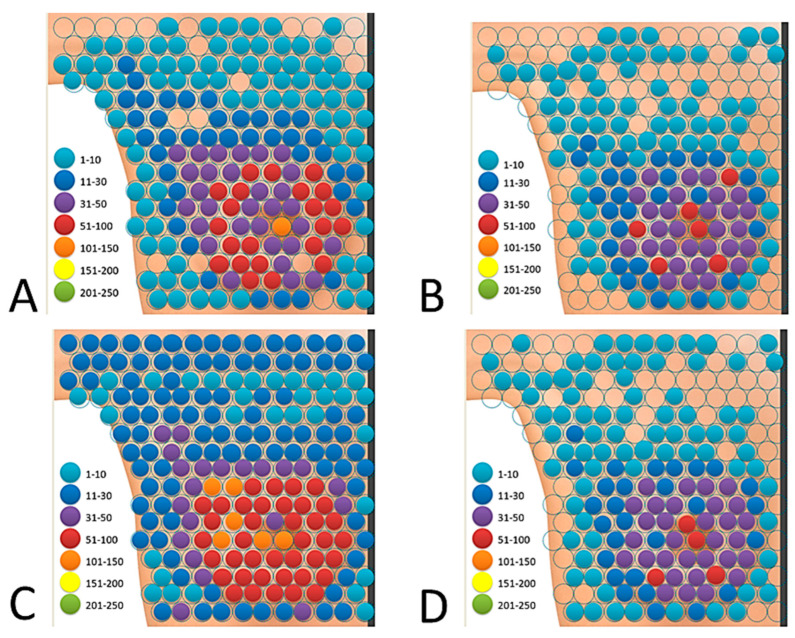
In the proprietary interactive tactile test “breast self-examination”, the distribution of the total marked area of the breast by all users in both groups: (**A**) the study group—Test I, (**B**) the control group—Test I, (**C**) the study group—Test II and (**D**) the control group—Test II.

The total amount of marked “breast” area differed between the study group and the control group. The central area of the mammary gland with the nipple turned out to be of greatest interest in both groups. A detailed analysis of all the points marked by the women allowed us to conclude that, in Test I, none of the groups was able to mark the entire breast area. In the study group, the users of the application marked a total of 178 points out of 200 possible, which is 89.0% of the “breast” area. After summing up the responses of 250 women from the study group, it turned out that 11.0% (22 areas) of the “breast” area was not marked by any of the examined women (Figure 6A, Table 5). In the study group, the nipple was marked by 54.4% (N = 136) of the women.

In the control group, a total of 137 points out of 200 were marked, which is 68.5% of the “breast” area. After summing up the measurements of 250 women from the control group, it turned out that 31.5% of the “breast” area (63 points) was not marked. Moreover, it was found that the most overlooked site in this group was the area marginal to the central part of the breast, including the armpit, as shown in Figure 6B.

In the control group, the nipple was marked by 28.4% (N = 71) of women. A summary of the frequency of areas marked by both groups is presented in Table 5.

**Figure 7:**
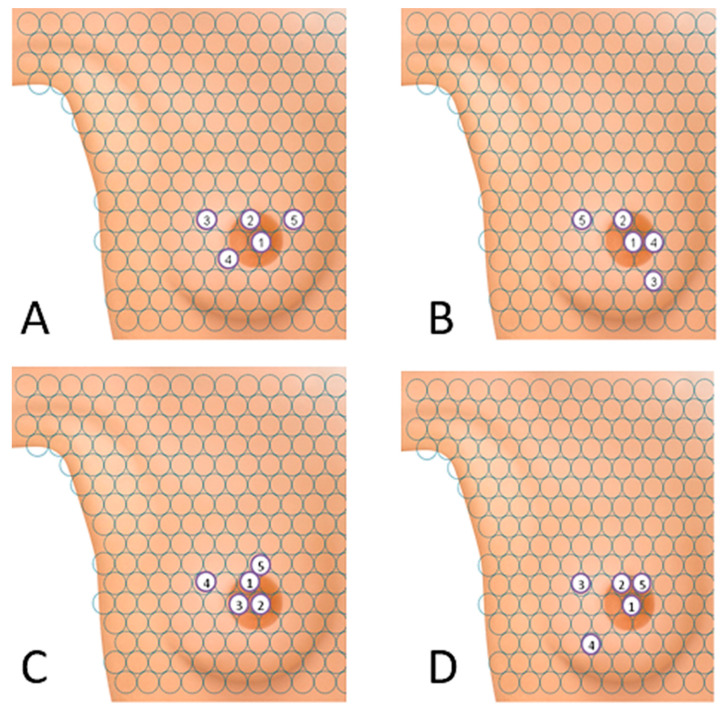
Points from which the breast examination was most often initiated in the proprietary interactive tactile test: (**A**) study group—Test I, (**B**) control group—Test I, (**C**) study group—Test II and (**D**) control group—Test II.

Women in both groups most often initiated the tactile test in the area of the nipple, Figure 7A,B. Most women in the study group (N = 103) indicated the nipple as the first place to be examined. The second most popular area was the area just above the nipple in Figure 7A.

In the control group, the order of the first two areas on the “breast” was similar to that in the study group, and 71 women chose the nipple as the first point of examination. The remaining three most popular areas were also in close proximity to the nipple, as shown in Figure 7B.

**Figure 8:**
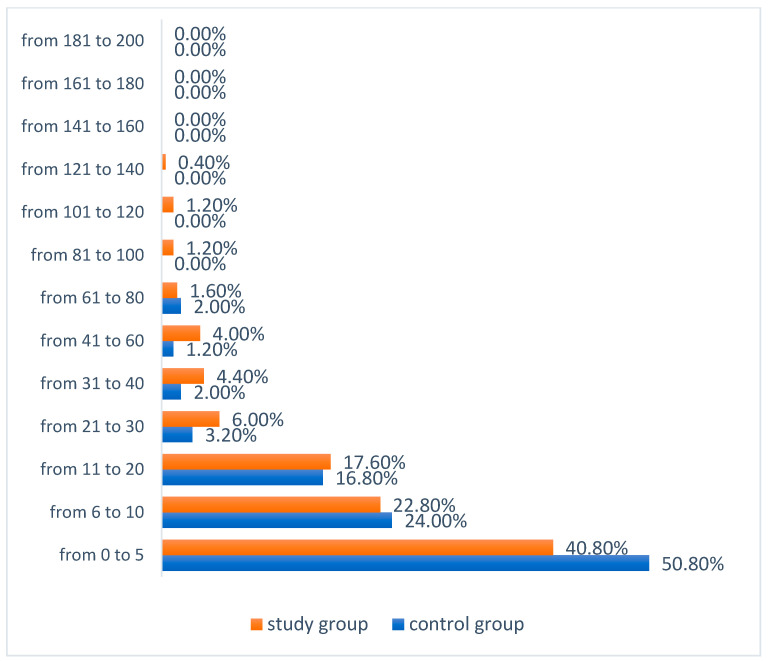
Number of app users marking a particular number of points in the tactile test—Test I.

In Test I, in both groups, the greatest number of users marked in the range of 0–5 points. In the study group it was significantly less women at 40.8% (N = 102) than in the control group at 50.80% (N = 127) (χ^2^ = 13.252; *df* = 6; *p* = 0.0392). In the control group, no one marked more than 100 points (more than 50% of the examined breast area), while in the study group, 1.6% (N = 4) marked more than half of the examined breast area, three women marked the area from 101 to 120 points (50.5–60% of the area) and one person marked 136 points (68% of the area), as shown in Figure 8. 

### 3.2. Results of Test II of the Proprietary Interactive Tactile Test

The results showed that in Test II up to 3 points were indicated by 15.8% of women (N = 79), more often by the subjects from the control group (28.8%). From 4 to 6 points were marked by a total of 23.6% of the respondents, also more often in the control group (32.8%, N = 82). From 7 to 13 points in the breast examination in Test II were marked by 29.6% of the respondents (N = 148), more often by people from the study group (N = 98, i.e., 39.2%). Similarly, above 13 points were marked more often by women in the study group (N = 109, i.e., 43.6%), and the total number of the respondents above 13 points were indicated by 31.0% of women (N = 155) (Table 6).

The total amount of marked “breast” area differed between the study group and the control group. Moreover, it was found that the central area of the mammary gland, mainly with the nipple, was of the greatest interest in both groups. A detailed analysis of all the points marked by the women allowed us to conclude that, in Test II, the study group was able to mark the entire breast surface. In the study group, the users of the application marked a total of 200 points out of 200 possible points, which is 100.0% of the “breast” area (Figure 6C, Table 7).

In the control group, a total of 143 points out of 200 were marked, which is 71.5% of the “breast” area. After summing up the measurements of 250 women from the control group, it turned out that 28.5% of the “breast” area (57 points) was not marked. Moreover, it was found that the marginal area was one of the most omitted places in the examination in this group in relation to the central part of the mammary gland and the upper inner quadrant (Figure 6D, Table 7).

As a result of the analysis of the research results, when starting the tactile test, the examined women in both groups most frequently chose the “breast” area near the nipple (Figure 7C,D). It was observed that in the study group the largest number of women (N = 57) indicated the area located on the areola just above the nipple as the first place of the study. The second most popular point was the nipple (N = 44) (Figure 7C).

In the control group, 51 women chose the nipple as the first point to be examined. The next two points were slightly outward from the nipple. The point chosen as the fifth in the sequence was on the areola, just above the nipple (Figure 7D).

The results of Test II showed a significant difference, people from the study group marked a larger area in the tactile test (χ^2^ = 99.733; *df* = 6; *p* < 0.0001).

**Figure 9:**
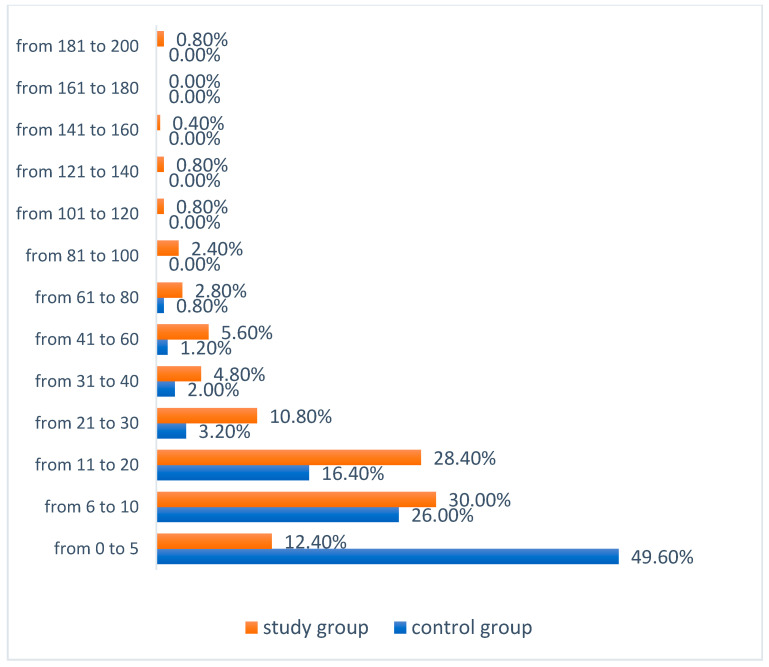
Number of application users marking a particular number of points in the tactile test—Test II.

In Test II, the greatest number of users from the control group marked the number of points in the range of 0–5 points at 49.6% (N = 124), while in the study group, 30.0% of women (N = 75) marked from 6 to 10 points, and in this group, 28.4% (N = 71) of the respondents chose from 11 to 20 points. In the control group, the highest score was 71 points. However, in the study group, 0.8% (N = 2) of women marked 200 points. More than 50% of the breast area was marked by seven people from the study group and five from the control group (Figure 9). Half of the study group obtained a result Me = 12.00. The minimum result in the study group was Min = 2.00, while the maximum result was Max = 200.00. Half of the control group obtained a result not lower than Me = 6.00. The minimum score in the control group was Min = 0.00, and the maximum score was Max = 71.00 (Table 8).

The results revealed that the application of the intervention significantly increased the ability to perform breast self-examination among the application users. Analyses were carried out on the number of points indicated in Tests I and II, the distribution of the total marked area of the breast by all users in both groups in Tests I and II and the number of points selected in the tactile test by individual users in Tests I and II. In all cases, there was an increase in the number of marked points. The differences between the number of points indicated in Tests I and the results of Test II were statistically significant (*p* < 0.0001)—3 pts. or less—was indicated more often in Test I (27.2%) than in Test II (15.8%). Meanwhile, 7–13 points or above 13 points were indicated in Test II more often than in Test I (Table 9).

Data analysis showed a reduction in the total amount of unexamined breast area in both groups in Test II compared to Test I. In total, women in the study group during Test II did not miss any point on the breast surface. In the control group, the total unexamined area decreased by 3.0% (Table 9).

**Figure 10:**
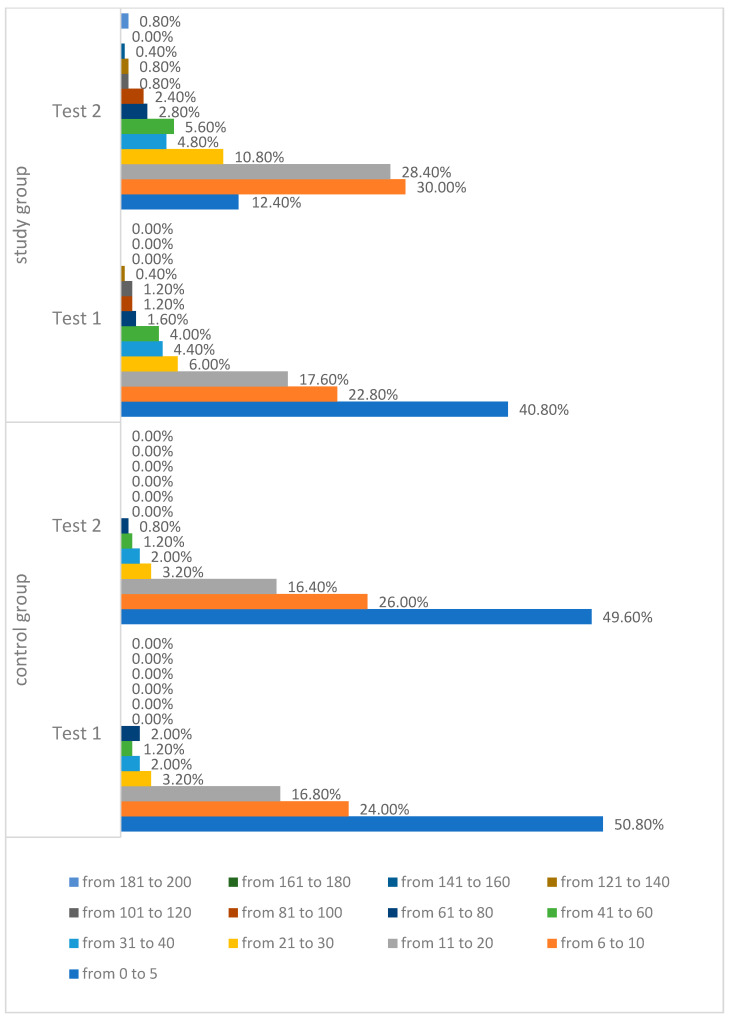
Proprietary interactive tactile test “breast self-examination”, number of points marked in the tactile test by individual users, Tests I and II.

It was also shown that the amount of the examined area of the breast (number of points) in the tactile test by individual application users increased between Tests I and II in both groups, which proves the validity of the effectiveness of the intervention. It was shown that the study group had a significantly larger area during Test II than the control group (χ^2^ = 53.448; *df* = 6; *p* < 0.0001) (Figure 10).

Multivariate linear regression was used to analyze the factors influencing the number of points in the breast self-examination. The explanatory variables were as follows: List of Health Criteria, Health Behavior Inventory, Generalized Self-Efficacy Scale, the Breast Cancer Risk Test and the proprietary questionnaire: Test your knowledge about breast cancer. The explained variable was the number of points indicated in the proprietary interactive tactile Breast self-examination test. The predictors were introduced into the model by the method of inputting the variables. The same calculation procedure was used for the number of points indicated in Tests I and II (Table 10)

The results revealed that the number of points indicated on the breast, both in Tests I and II, depended solely on women’s knowledge about breast cancer (*p* < 0.001). The higher the knowledge of breast cancer, the greater the number of points indicated on the breast in Test I (β = 0.40; *p* < 0.0001) and in Test II (β = 0.35; *p* < 0.0001) (Table 10).

The data analysis showed the existence of statistically significant correlations, which indicate that there are relationships between the level of knowledge and the number of points marked in the tactile test both in Test I and II. The study indicated that with the increase in the result of the level of knowledge about cancer (Test I), the frequency of marked points in the tactile test increased both in Test I (χ^2^ = 103.684, *df* = 12, *p* < 0.001) and in Test II (χ^2^ = 95.832, *df* = 12, *p* < 0.001) (Table 11).

The cross-analysis of the obtained data showed a statistically significant correlation, which links the level of knowledge obtained in Test II with the number of points tested in the original interactive tactile test—“breast self-examination” (Test I). The observed correlation informs that the more points marked on the “breast” in the tactile test during Test I, the higher the level of knowledge in Test II (χ^2^ = 59.651, *df* = 12, *p* < 0.001) (Table 12).

## 4. Discussion

Breast self-examination (BSE) is a key element in promoting knowledge about breast neoplasms [3], and numerous studies confirm its important role in the early diagnosis of breast cancer [4,5,6,7,8,9]. BSE is considered an important first step to encourage women to be actively responsible for their own health [8,10]. This examination is non-invasive, easy to perform, cost-free and regularly performed in conjunction with women’s self-awareness about the structure of their own breasts and cyclical changes in them is the key to detect breast cancer. It is especially important in the case of young women and those in high risk of this disease [11]. Many women do not perform BSE due to the lack of knowledge and skills in this respect, and many women who perform BSE do it irregularly and incorrectly [12]. From the point of view of the effectiveness of breast self-examination, it is extremely important to examine the entire surface of the mammary gland, including the axillary and subclavian regions. In our study in Test I, it was shown that on average in both groups, women marked only 6% of the breast area that should be examined.

Therefore, health education in the field of breast cancer prevention and the technique of breast self-examination are required in order to reach a wide group of young women. In addition to information on the BSE technique, it is important to try to assess women’s skills in this area. In the proprietary app “Exam oneself”, the following parameters were assessed: evaluation of the marked area, determination of the most frequently marked and omitted places on the breast and areas on the breast model from which the examination was most often started. When performing breast self-examination, it is important to perform three-stage compression in the examined area of the breast gland during the examination. In the “Exam oneself” app, it was not possible to determine whether the person performing the test uses three-stage pressure. Such a solution is possible, but on a specially constructed device equipped with pressure sensors, as currently available mobile devices (smartphones, tablets) do not have such sensors as standard. Hence, the limitation of this project is the lack of evaluation of the use of three-stage compression. However, application users were informed about this issue.

The method of providing information in the study was intervention and personalized education, thanks to which the user was presented with the information in terms of which a knowledge deficit was found in Test I of the knowledge test. The results of this study confirm that a mobile medical app containing a conditional instruction with assigned points may contribute to the improvement of the ability to properly perform breast self-examination. 

As the results of our study have shown, the use of the intervention significantly influences the development of the ability to perform breast self-examination, and personalization significantly increases this skill among application users. In Test II, there was an increase (improvement in the ability to perform breast self-examination) compared to Test I in terms of the number of points indicated and the total amount of unmarked breast area by all users in both groups, while the women from the study group did not omit any field during Test II on the examined surface of the breast. The number of points marked in the tactile test by individual users in both groups also improved. It has been shown that in the study group, significantly more points were marked during Test II than in the control group. Undoubtedly, such a result results from the introduced personalized education, which was performed among women from the study group. Similar results in terms of the effectiveness of the use of mobile medical applications were obtained in the studies by Pruthi et al., in which 60 consultations conducted via the application in the field of breast cancer were assessed on a sample of 15 women. Overall, 98% of the respondents showed satisfaction with the consultations [13]. Similar results were demonstrated by Morgan et al. in 25 patients diagnosed with early breast cancer who participated in the study. The respondents were provided with tablets equipped with an educational program in the field of knowledge about complementary treatment. It has been shown that education based on the use of mobile devices can be a feasible and effective method of educating patients [14]. A Chinese study by Zhu et al. also demonstrated the usefulness of an individually tailored mobile application to support women with breast cancer undergoing chemotherapy [15]. In turn, the study by O’Reilly et al. provided evidence that the use of personalization in the form of an application for so-called cancer survivors may increase the level of their daily physical activity [16]. Mobile interventions tailored to individual people are definitely more effective than those that are used in the same form for all [17].

The positive impact of education interventions on BSE implementation has been demonstrated in studies conducted in Saudi Arabia [18,19,20,21]. In studies by Tuna et al., the rate of systematic breast self-examination in women was 30.8% before the intervention, and after the intervention it increased to 47.8% [22]. Kissal and Kartal also believe that an individual approach to education can be more effective in learning BSE [23]. In contrast, Malak and Dicle believe that such education contributes to the increase in BSE performance by individuals but does not necessarily mean that the test will be properly performed [24].

The results of the studies also indicate the legitimacy of using interventions in the mobile form. The results of the study by Sahu et al. on the role of mobile technology in the implementation of health education programs in Asian and African countries (Philippines, China, Kenya, South Korea, Taiwan and India) showed that mobile technology contributed to the improvement of the health of chronically ill patients with diabetes, heart disease and arterial hypertension; there was also an improvement in the prevention of breast cancer [25]. The apps are successfully used in patients with breast cancer during adjuvant treatment, as well as among people who have recovered, in order to prevent relapse [26]. An innovative approach are solutions used in apps resembling a video game, increasing the motivation to take up physical activity, thanks to which the application user scores points or levels adequately to the way the application is used [27]. The study by McCarroll et al. confirms the legitimacy of using the medical application in changing the lifestyle in patients treated for breast and uterine cancer. The results show that people using the app showed a significant decrease in body weight [28].

The study attempted to identify important factors influencing the performance of the tactile test. An attempt was also made to link the GSES scale relating to the general belief of an individual about the effectiveness of coping with difficult situations and obstacles with the results of the author’s interactive tactile test. However, no statistically significant relationship was found between the tactile test results and self-efficacy. Perhaps it was due to the fact that the surveyed women had little age diversity, as 77.8% of the respondents were under 30 years of age.

Interesting insights on the GSES scale are provided by the results of a review of 24 studies on self-efficacy in women with breast cancer, which showed that self-efficacy in breast cancer is a key element in improving goal-directed behavior in patients and should be supported by healthcare professionals and family members [29]. A positive correlation was also shown in studies assessing the level of psychological resistance of women after breast cancer surgery and the total GSES results [30]. Positive relationships were also observed in the study assessing learning ability, metacognitive ability and self-efficacy in a sample of nursing students. The authors of these studies unanimously recommend carrying out further analyzes using this scale. We also consider it to be justified to conduct further research using the GSES and the tactile test on a larger group of women in various age groups.

From the point of view of the purposefulness of breast self-examination, predictors influencing the performance of the examination are important. Numerous studies confirm that the most frequently reported obstacle in performing breast self-examination is the lack of knowledge in this field [31,32,33]. Also in our research it was shown that knowledge turned out to be the main predictor influencing the number of marked points in the tactile test. The results of our study showed the existence of a relationship between the level of knowledge and the number of marked points in the tactile test. The number of correctly indicated points on the breast in the tactile test, both in Test I and in Test II, depended solely on the women’s knowledge (*p* < 0.001). It is also interesting that the more points marked on “breasts” in the tactile test during Test I, the higher was the level of women’s knowledge in Test II (χ^2^ = 59.651, *df* = 12, *p* < 0.001). However, the lower the level of knowledge in Test II, the smaller the number of marked points on the “breast” in the tactile test (measurement II) (χ^2^ = 24.073, *df* = 9, *p* = 0.004). The obtained results are in line with those obtained in a college study in the southeast United States by the team of Guilford et al., in which it was shown that knowledge of breast cancer was significantly correlated with breast self-examination [34], as well as Iranian studies conducted on 334 students from the Urmia Medical University in northwestern Iran [G]. This study showed that the high level of knowledge compared to the low level of knowledge (OR = 5.51, 95% CI = (1.79–16.86)) and education were predictors of BSE effectiveness (*p* < 0.05). Additionally, other authors have shown that the BSE performance rate is definitely higher in people with a good or high level of knowledge in this area [35,36]. On the other hand, no relationship between knowledge and practice of BSE was demonstrated by Ghodsi and Hojjatoleslami [37].

Algorithms are used successfully in medicine, including the diagnosis and treatment of many diseases. Machine learning is widely used in breast cancer classification. It provides high classification accuracy and effective diagnostic possibilities. The developed automatic classification algorithm for the identification of neoplasms in the mammary gland on dedicated breast CT images showed high accuracy in the classification of various types of tissues [38]. Research into the support vector machines (SVM) algorithm for breast cancer diagnosis has also shown greater accuracy in breast cancer diagnosis [39].

## 5. Conclusions

1. A mobile medical application containing a conditional instruction with assigned points for breast self-examination contributed to the increase in the ability to properly perform the breast self-examination technique.
2. There is a need to improve the mobile tool with a module for the verification of the skills of the three-stage compression of the examined breast.
3. Educational mobile medical applications on breast cancer prevention can be helpful in solving the public health problem related to breast cancer, especially during the pandemic.

## Figures and Tables

**Table 1:** Characteristics of the studied population.

			Group			Test Result
			Study	Control	Total	
Age	30 years or below	N	202	187	389	χ^2^ = 1.988 *df* = 4 *p* = 0.738
		%	80.8	74.8	77.8	
	31–40 yrs	N	17	24	41	
		%	6.8	9.0	7.9	
	41–50 yrs	N	21	23	44	
		%	8.4	9.2	8.8	
	51–60 yrs	N	10	14	24	
		%	4.0	5.6	4.8	
	Over 60 yrs	N	1	2	3	
		%	0.4	0.8	0.6	
Place of residence	Rural area	N	137	128	265	χ^2^ = 1.584 *df* = 2 *p* = 0.453
		%	54.8	51.2	53	
	Rzeszów	N	54	66	120	
		%	21.6	26.4	24.0	
	Another city in the Podkarpackie Province	N	59	56	115	
		%	23.6	22.4	23.0	
Education	Primary/lower secondary	N	18	14	32	χ^2^ = 0.590 *df* = 2 *p* = 0.745
		%	7.2	5.6	6.4	
	Secondary	N	146	151	297	
		%	58.4	60.4	59.4	
	Higher	N	86	85	171	
		%	34.4	34,0	34.2	
Work performed	Physical	N	52	48	100	χ^2^ = 0.608 *df* = 3 *p* = 0.895
		%	20.8	19.2	40.0	
	Mental	N	66	69	135	
		%	26.4	27.6	27.0	
	Do not work	N	33	29	62	
		%	13.2	11.6	12.4	
	Do not work/study	N	99	104	203	
		%	39.6	41.6	40.6	

χ^2^—test statistics; *df*—degrees of freedom; *p*—statistical significance.

**Table 2:** Number of points marked on the breast model—Test I.

Variable			Group			Test Result
			Study	Controls	Total	
Number of points marked—Test I	0–3	N	64	72	136	χ^2^ = 14.535 *df* = 3 *p* = 0.002
		%	25.6	28.8	27.2	
	4–6	N	53	82	135	
		%	21.2	32.8	27.0	
	7–13	N	57	50	107	
		%	22.8	20.0	21.4	
	More than 13	N	76	46	122	
		%	30.4	18.4	24.4	
Total		N	250	250	500	
		%	100.0	100.0	100.0	

χ^2^—test statistics; *df*—degrees of freedom; *p*—statistical significance.

**Table 3:** Number of points marked in the tactile test—Test I.

Group		Test I Number of Points Examined
study	Mean	14.86
	SD	21.60
	Median	7.00
	Min	0.00
	Max	136.00
	N	250
controls	Mean	9.14
	SD	11.71
	Median	5.00
	Min	0.00
	Max	71.00
	N	250
Total	Mean	12.00
	SD	17.59
	Median	6.00
	Min	0.00
	Max	136.00
	N	500

SD—standard deviation, Min—minimum, Max—maximum.

**Table 4:** Mann–Whitney U Test for independent trials—Test I.

				Descriptive Statistics		
		U	*p*	Min.	Max.	Me
Group	Breast self-examination—Test I	26,873.50	0.007			
	study			0.00	136.00	7.00
	control			0.00	71.00	5.00

U—test statistic, *p*—statistical significance, Me—median, Min—minimal result, Max—maximum result.

**Table 5:** In the proprietary interactive tactile test “breast self-examination”, the amount of marked area—Test I.

	Marked Area (Number of Points)		Not Marked Area (Number of Points)		
	N	%	N	%	
Study group	178	89.0	22	11.0	χ^2^ = 21.440 *df* = 1 *p* < 0.003
Control group	137	68.5	63	31.5	

χ^2^—test statistics; *df*—degrees of freedom; *p*—statistical significance.

**Table 6:** Number of points subjected to breast examination—Test II.

			Group			Test Result
			Study	Control	Total	
Number of points marked—Test II	Up to 3 pts	N	7	72	79	χ^2^ = 112.587 *df* = 3 *p* < 0.001
		%	2.8	28.8	15.8	
	4–6 pts	N	36	82	118	
		%	14.4	32.8	23.6	
	7–13 pts	N	98	50	148	
		%	39.2	20.0	29.6	
	More than 13 pts	N	109	46	155	
		%	43.6	18.4	31.0	
Total		N	250	250	500	
		%	100.0	100.0	100.0	

χ^2^—test statistics; *df*—degrees of freedom; *p*—statistical significance.

**Table 7:** Proprietary interactive tactile test “breast self-examination”, and the amount of marked area—Test II.

	Marked Area (Number of Points)		Not Marked Area (Number of Points)		
	N	%	N	%	
Study group	200	100.0	0	0	χ^2^ = 17.02 *df* = 1 *p* < 0.001
Control group	143	71.5	57	28.5	

χ^2^—test statistics; *df*—degrees of freedom; *p*—statistical significance.

**Table 8:** Number of points marked in the tactile test—Test I and II.

Group		Test I Number of Points Examined	Test II Number of Points Examined
Study	Mean	14.86	22.10
	SD	21.60	28.53
	Median	7.00	12.00
	Min	0.00	2.00
	Max	136.00	200.00
	N	250	250
Control	Mean	9.14	9.10
	SD	11.71	11.75
	Median	5.00	6.00
	Min	0.00	0.00
	Max	71.00	71.00
	N	250	250
Total	Mean	12.00	15.60
	SD	17.59	22.74
	Median	6.00	8.00
	Min	0.00	0.00
	Max	136.00	200.00
	N	500	500

SD—standard deviation, Min—minimum, Max—maximum.

**Table 9:** Proprietary interactive tactile test “breast self-examination”, and the total number of marked points—Test I and II.

	Marked Area (Number of Points)		Marked Area (Number of Points)		Marked Area (Number of Points)		Marked Area (Number of Points)		Test Result
	Test I	Test II	Test I	Test II	Test I	Test II	Test I	Test II	
	N	N	%	%	N	N	%	%	
The study group	178	200	89.0	100.0	22	0	11.0	0.0	χ^2^ = 0.218 *df* =1 *p* = 0.6401 (marked area) χ^2^ = 15.53 *df* =1 *p* < 0.001 (not marked area)
The control group	137	143	68.5	71.5	63	57	31.5	28.5	

**Table 10:** Influence of selected factors on the number of points indicated in breast self-examination—multivariate linear regression using the variable input method.

Model		Non-Standardized Coefficients		Standardized Coefficients	*t*	*p*
		B	Standard Error	Beta		
The number of points indicated in the proprietary interactive tactile test: Breast self-examination Test I	Generalized Self-Efficacy Scale (GSES)	−0.32	0.19	−0.07	−1.73	0.0841
	Proprietary questionnaire: Test your knowledge about breast cancer	1.61	0.16	0.40	9.77	*p* < 0.001
The number of points indicated in the proprietary interactive tactile test: Breast self-examination Test II	Generalized Self-Efficacy Scale (GSES)	−0.39	0.25	−0.07	−1.56	0.1188
	Proprietary questionnaire: Test your knowledge about breast cancer	1.60	0.19	0.35	8.31	*p* < 0.001

**Table 11:** The relationship between the level of knowledge (Test I) and the number of marked points in the tactile test (Tests I and II).

-			Knowledge—Test I—Ranges					Test Result
			Very Low	Low	Average	High	Very High	
the proprietary interactive tactile test: Breast self-examination (Test I)	3 pts or less	N	41	70	23	2	0	χ^2^ = 103.684 *p* < 0.001
		%	51.3	32.1	16.1	4.3	0.0	
	4–6 pts	N	20	70	37	6	2	
		%	25.0	32.1	25.9	12.8	16.7	
	7–13 pts	N	15	44	34	9	5	
		%	18.8	20.2	23.8	19.1	41.7	
	More than. 13 pts	N	4	34	49	30	5	
		%	5.0	15.6	34.3	63.8	41.7	
the proprietary interactive tactile test: Breast self-examination (Test II)	3 pts or less	N	23	50	6	0	0	χ^2^ = 95.832 *p* < 0.001
		%	28.8	22.9	4.2	0.0	0.0	
	4–6 pts	N	24	62	30	2	0	
		%	30.0	28.4	21.0	4.3	0.0	
	7–13 pts	N	24	55	50	13	6	
		%	30.0	25.2	35.0	27.7	50.0	
	More than 13 pts	N	9	51	57	32	6	
		%	11.3	23.4	39.9	68.1	50.0	

χ^2^—test statistics; *p*—statistical significance.

**Table 12:** Knowledge in Test II and the number of tested points in the proprietary interactive tactile test in Tests I and II.

			Knowledge—Test II—Ranges					Test Result
			Very Low	Low	Average	High	Very High	
The Proprietary interactive tactile test: “breast self-examination” (Test I)	3 pts or less	N	17	49	35	32	3	χ^2^ = 59.651 *df* = 12 *p* < 0.001
		%	42.5	37.1	26.3	20.0	8.6	
	4–6 pts	N	14	43	41	32	5	
		%	35.0	32.6	30.8	20.0	14.3	
	7–13 pts	N	7	23	28	41	8	
		%	17.5	17.4	21.1	25.6	22.9	
	More than. 13 pts	N	2	17	29	55	19	
		%	5.0	12.9	21.8	34.4	54.3	
The Proprietary interactive tactile test: “breast self-examination” (Test II)	3 pts or less	N	17	47	11	4	0	χ^2^ = 159.437 *df* =12 *p* < 0.001
		%	42.5	35.6	8.3	2.5	0.0	
	4–6 pts	N	14	42	40	20	2	
		%	35.0	31.8	30.1	12.5	5.7	
	7–13 pts	N	7	24	46	60	11	
		%	17.5	18.2	34.6	37.5	31.4	
	More than. 13 pts	N	2	19	36	76	22	
		%	5.0	14.4	27.1	47.5	62.9	

χ^2^—test statistics; *df*—degrees of freedom; *p*—statistical significance.

## Data Availability

The authors can be contacted for information on the dataset.

## Supplementary Materials

The following supporting information can be downloaded at: https://www.mdpi.com/article/10.3390/ijerph19084482/s1.
