# Supplementary material for: Application of Personalized Education in the Mobile Medical App for Breast Self-Examination
Source: Int J Environ Res Public Health. 2022 Apr 8;19(8):4482. doi: 10.3390/ijerph19084482 (PMC9032731; doi:10.3390/ijerph19084482)

Supplementary A proprietary questionnaire: Test your knowledge about breast cancer

1. Does breast cancer affect only women??
    a. Yes
    b. No
    c. I don’t know

2. Is breast cancer curable at an early stage?

    a. Yes
    b. No
    c. I don’t know

3. What methods of breast cancer detection performed in women of your age do you know? *(more than 1 answer can be selected)*

    a. Breast self-examination
    b. Mammography
    c. Magnetic resonance imaging
    d. Chest X-ray
    e. Breast biopsy
    f. Computed tomography
    g. breast ultrasound

4. Can a woman detect breast cancer by herself??
    a. Yes
    b. No
    c. I don’t know

5. How often should you self-examine breasts?
    a. Once a month
    b. Once a year
    c. Once every two years
    d. Only when I have a problem

6. Breast self-examination is about:
    a. Inspection of your breasts
    b. Inspection and palpation of your breast
    c. Only palpation of your breast
    d. Examination by a physician

7. When is the best time to do breast self-examination?
    a. Just before your period
    b. 4-7 days after menstruation
    c. No matter when
    d. During menstruation

8. With part of the hand is used for breast examination?

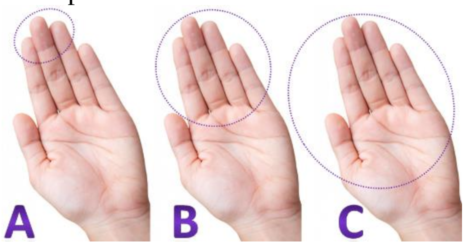

9. When performing breast self-examination, what part of the breast surface should be examined?
    a. A
    b. B
    c. C
    d. D

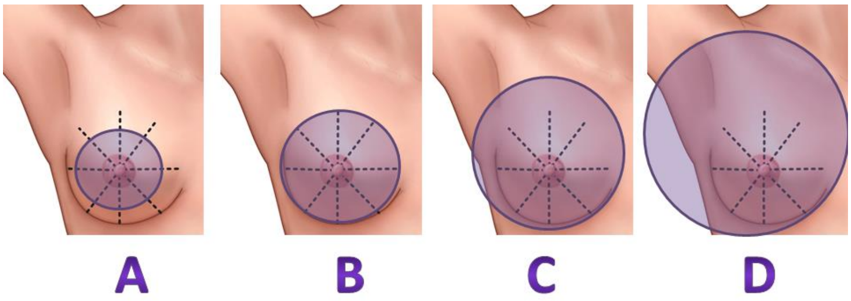

10. What factors can cause breast cancer? *(more than 1 answer can be selected)*
    a. Stress
    b. Environment
    c. Smoking cigarettes or alcohol
    d. Family history of breast cancer
    e. Obesity
    f. First menstruation at an early age
    g. Poor nutrition
    h. Age
    i. Childlessness
    j. Carrier of mutations of certain genes (BRCA1 / BRCA2)
    k. Badly fitted bra
    l. Menopause in late age
    m. Long-term use of oral contraceptives

11. What are the symptoms of breast cancer?
    a. A lump, hardness, or thickening in the breast
    b. Nipple retracted / collapsed
    c. Pits / concavities
    d. Skin ulceration
    e. Redness of the skin
    f. Pain in the breast
    g. Discharge from the nipple
    h. Enlargement of axillary lymph nodes
    i. Breast asymmetry (differences in the shape and size of the breasts)
    j. "Orange peel" changes on the surface of the breast

Supplementary Proprietary interactive tactile test

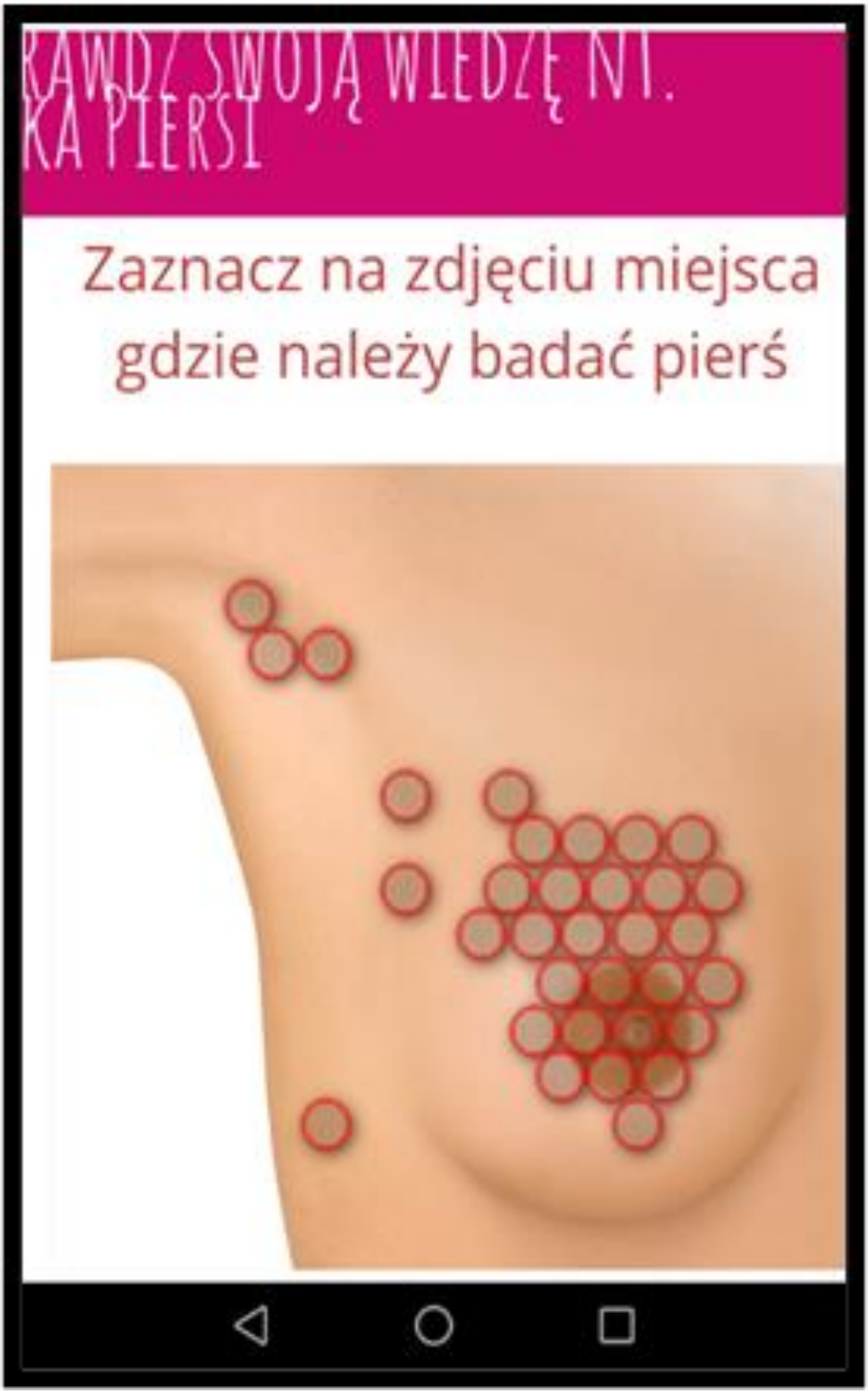

Supplement: Supplementary file 1 [file ijerph-19-04482-s001.zip › ijerph-1601698-supplementary.pdf]
